# Multimodality Imaging in Arrhythmogenic Left Ventricular Cardiomyopathy

**DOI:** 10.3390/jcm12041568

**Published:** 2023-02-16

**Authors:** Emanuele Monda, Marta Rubino, Giuseppe Palmiero, Federica Verrillo, Michele Lioncino, Gaetano Diana, Annapaola Cirillo, Adelaide Fusco, Francesca Dongiglio, Martina Caiazza, Ippolita Altobelli, Alfredo Mauriello, Natale Guarnaccia, Alessandra Scatteia, Arturo Cesaro, Giuseppe Pacileo, Berardo Sarubbi, Giulia Frisso, Barbara Bauce, Antonello D’Andrea, Santo Dellegrottaglie, Maria Giovanna Russo, Paolo Calabrò, Giuseppe Limongelli

**Affiliations:** 1 Inherited and Rare Cardiovascular Diseases, Department of Translational Medical Sciences, University of Campania “Luigi Vanvitelli”, Monaldi Hospital, 80131 Naples, Italy; 2 Institute of Cardiovascular Sciences, University College of London and St. Bartholomew’s Hospital, London WC1E 6DD, UK; 3 Division of Cardiology, Clinica Villa dei Fiori, 80011 Acerra, Italy; 4 Dipartimento di Medicina Molecolare e Biotecnologie Mediche, University of Naples “Federico II”, 80138 Naples, Italy; 5 Department of Cardiac, Thoracic and Vascular Sciences and Public Health, University of Padova, 35128 Padova, Italy; 6 Department of Cardiology and Intensive Coronary Care, Umberto I Hospital, 84014 Nocera Inferiore, Italy

**Keywords:** arrhythmogenic cardiomyopathy, multimodality imaging, echocardiography, cardiac magnetic resonance, positron emission tomography

## Abstract

The term arrhythmogenic cardiomyopathy (ACM) describes a large spectrum of myocardial diseases characterized by progressive fibrotic or fibrofatty replacement, which gives the substrate for the occurrence of ventricular tachyarrhythmias and the development of ventricular dysfunction. This condition may exclusively affect the left ventricle, leading to the introduction of the term arrhythmogenic left ventricular cardiomyopathy (ALVC). The clinical features of ALVC are progressive fibrotic replacement with the absence or mild dilation of the LV and the occurrence of ventricular arrhythmias within the left ventricle. In 2019, the diagnostic criteria for the diagnosis of ALVC, based on family history and clinical, electrocardiographic, and imaging features, have been proposed. However, since the significant clinical and imaging overlap with other cardiac diseases, genetic testing with the demonstration of a pathogenic variant in an ACM-related gene is required for diagnostic confirmation. In ALVC, the multimodality imaging approach comprises different imaging techniques, such as echocardiography, cardiac magnetic resonance, and cardiac nuclear imaging. It provides essential information for the diagnosis, differential diagnosis, sudden cardiac death risk stratification, and management purposes. This review aims to elucidate the current role of the different multimodality imaging techniques in patients with ALVC.

## 1. Background

The term arrhythmogenic cardiomyopathy (ACM) describes a large spectrum of myocardial diseases characterized by progressive fibrotic or fibrofatty replacement, which gives the substrate for the occurrence of ventricular tachyarrhythmias and the development of ventricular dysfunction [1].

In the pregenetic era, ACM was originally described as a myocardial disease exclusively involving the right ventricle (RV), and the term arrhythmogenic RV dysplasia/cardiomyopathy (ARVD/ARVC) has been used [2,3]. However, with a large introduction of cardiac magnetic resonance (CMR) and genotype–phenotype studies, it was observed that the left ventricle (LV) is commonly involved in fibrotic replacement, changing the paradigm of the disease. In addition, it was observed that, in some cases, it is possible that this pathophysiological process exclusively affects the LV, leading to the introduction of the term arrhythmogenic LV cardiomyopathy (ALVC) [4,5,6].

The clinical features of ALVC are progressive fibrotic replacement with the absence or mild dilation of the LV and the occurrence of ventricular arrhythmias within the LV [6]. Sudden cardiac death (SCD) could be the first manifestation of the disease [7]. In addition, the genetic background of ALVC is significantly different from those of ARVC. ALVC is mainly caused by pathogenic variants in non-desmosomal genes (i.e., in the gene encoding for the ion channel, sarcomeric, cytoskeletric, or mitochondrial proteins), similar to those responsible for dilated cardiomyopathy (DCM) [8,9,10]. It has been proposed that ALVC and DCM represent the two extremes of a large spectrum of disease, and the term hypokinetic non-dilated cardiomyopathy (HNDCM) is commonly used to describe a clinical phenotype characterized by LV dysfunction with or without the occurrence of arrhythmias and no LV dilatation [11].

The original criteria for the diagnosis of ACM, the 2010 task force criteria, are exclusively focused on the RV [12]. Thus, in 2019, the diagnostic criteria for the diagnosis of ALVC have been proposed (i.e., the international criteria or the “Padua criteria”) [13] (Table 1). The criteria have been focused on electrocardiographic changes associated with LV involvement (e.g., low QRS voltages in the limb leads), ventricular arrhythmias, or repetitive premature ventricular complexes with a right bundle branch block pattern (originating from the LV), and structural and functional imaging features consistent with LV involvement. CMR has a dominant role in ALVC since the demonstration of fibrosis (expressed as late gadolinium enhancement [LGE]) is a major diagnostic criterion for the diagnosis. Finally, for diagnostic confirmation, genetic testing with the demonstration of a pathogenic variant in an ACM-related gene is required [13,14].

Several genetic and non-genetic conditions exhibit significant clinical and overlapping imaging features with ALVC, such as DCM, myocarditis, or cardiac sarcoidosis [14].

In ALVC, the multimodality imaging approach comprises different imaging techniques, such as echocardiography, CMR, and cardiac nuclear imaging (CNI). It provides essential information for the diagnosis, differential diagnosis, sudden cardiac death risk stratification, and management purposes.

This review aims to elucidate the current role of the different multimodality imaging techniques in patients with ALVC.

## 2. Echocardiography

According to the 2020 international criteria, the diagnosis of ACM is focused on a multiparametric approach involving morphological ventricular anomalies, structural myocardial tissue changes, depolarization and repolarization electrocardiographic alterations, arrhythmias, family history, and genetic background [13]. In ALVC, the diagnosis needs at least one morpho-functional or structural criterion plus a pathogenic mutation because of the lack of a disease-specific phenotype [14].

Among these proposed diagnostic criteria, there are two minor imaging findings detectable through echocardiography: global LV systolic dysfunction with or without LV dilation; regional LV hypokinesia or akinesia of the free wall, septum, or both [13]. The most common clinical phenotype is that of an HNDCM with absent or mild dilation. The ventricular function deteriorates progressively as a result of the involvement of multiple LV segments. These structural abnormalities correlate with genetic mutations and the disease stage. Current genotype–phenotype correlations suggest that ACM with an intense arrhythmic burden may be associated either with mild ventricular dysfunction or with severe impairment of LV function. Of note, one of the most studied genes is lamin A/C (LMNA), where life-threatening ventricular arrhythmias can occur, even without significant LV impairment [15,16].

Echocardiography is often the first-line imaging technique since it is largely available, non-invasive, and easily repeatable. It could provide information on the cardiac phenotype, suggesting the disease etiology, morphology, hemodynamics, and disease severity.

LV systolic dysfunction can be detected by echocardiography as the reduction of the LV ejection fraction (LVEF) or in global longitudinal strain (GLS). Indeed, speckle-tracking echocardiography represents an indirect evaluation tool of systolic function, providing information about active myocardial deformation [17]. Furthermore, mechanical dispersion, calculated as the standard deviation of the time-to-peak regional negative strain, adds information about the extension of the myocardial damage and arrhythmic risk [18]. For this reason, an abnormal GLS and a higher mechanical dispersion suggest a larger injury that promotes severe fibrosis and potential re-entry circuits. Moreover, the speckle-tracking technique is useful for highlighting regional LV involvement without a reduced global function because of segmental disease progression [17,18].

As discussed below, the LVEF is the main parameter used to stratify the SCD risk of individual ALVC patients. Next to the LVEF, it has been proposed that LV mechanical dispersion could improve risk stratification in patients with ALVC and *PLN* mutation. The authors investigated 243 *PLN R14del* mutation carriers, categorized into three groups, according to the LVEF and the LV mechanical dispersion using the “45/45” rule [19]. Patients with an LVEF < 45% exhibited a high rate of ventricular arrhythmias and were classified as high-risk patients. Patients with preserved LV function and normal LV mechanical dispersion (an LVEF > 45% and an LVMD of <45 ms) experienced rare episodes of ventricular arrhythmias and were classified as low-risk patients. Finally, those with a preserved LVEF but with mechanical LV dysfunction (an LVEF > 45% and an LVMD > 45 ms) exhibited an intermediate risk for the development of ventricular arrhythmias, falling into a “grey zone” where a multiparametric assessment for SCD risk prediction is required [19].

Despite the potential role of echocardiography for the diagnosis of ALVC, tissue characterization findings by CMR represent the standard tool for disease confirmation in all patients with LV involvement [13,14].

## 3. Cardiac Magnetic Resonance

In recent years, CMR has gained a dominant role in the diagnosis of ACM, given its high accuracy and reproducibility in evaluating the chamber volume and the possibility of providing tissue characterization. Compared with echocardiography, its higher accuracy in determining LV mass, LV volumes, and LVEF is related to high contrast and spatial resolution, thus representing the gold standard technique for chamber quantification. In addition, CMR exhibits higher inter- and intra-observer analysis reproducibility. However, it is less available, exhibits higher costs, and presents absolute (e.g., an allergy to the gadolinium contrast and non-CMR compatible implants/foreign bodies) and relative contraindications (e.g., advanced renal disease and pregnancy), which limit its use in some cases.

**Figure 1:**
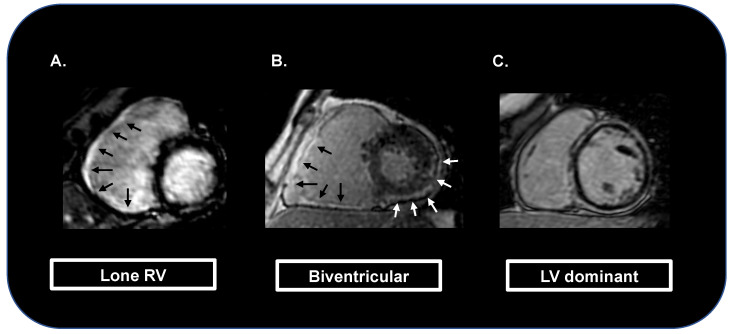
Cardiac magnetic resonance images showing myocardial fibrosis (as late gadolinium enhancement areas) limited to the right ventricle (black arrows; **A**), also extended to the left ventricle (white arrows; **B**), or with dominant left ventricular involvement («ring-like» late gadolinium enhancement pattern; **C**).

As mentioned above, the 2010 task force criteria for the diagnosis of ACM were focused on right ventricular involvement [12]. Myocardial tissue characterization was only based on endomyocardial biopsy samples, no diagnostic criteria for ALVC were provided, and the potential capacity of CMR in detecting tissue myocardial fibrosis using LGE was not mentioned [12]. In contrast, the 2020 international criteria give a dominant role to CMR. In particular, the identification of LGE in more than one myocardial segment is considered a major diagnostic criterion for the diagnosis of ACM [13,14] (Figure 1).

CMR study protocols for the suspicion of ALVC should include cine sequences for the assessment of the volumes, kinesis, and function of both the LV and RV, T1-weighted images to identify fatty infiltration, and LGE sequences for the detection of myocardial fibrosis.

In ACM, the progression of LV fibrotic replacement occurs from the subepicardial to the subendocardial layers. Thus, the final stage is the transmural fibrotic lesion, localized in single or multiple segments and leading to extensive wall thinning [20]. The subendocardial layer has a central role in wall contractility, thus explaining the lack or the limited presence of wall motion abnormalities in patients with ACM. Myocardial LV fat infiltration is easily detected, given the greater wall thickness than the RV, and exhibits a specific “bite-like” pattern [21].

The most common pattern of fatty infiltration and LGE distribution is at the level of the basal inferolateral and anterolateral walls, followed by the mid-inferoseptal, inferolateral, and anterolateral walls [22]. Native T1 mapping and extra-cellular volume (ECV) quantification are CMR sequences able to detect an increase in the interstitial space. It has been observed that native T1 values are higher in genotype-positive ACM patients than in the controls, probably related to the LV fibrosis observed in these individuals [23].

T2 sequences are generally not recommended for the diagnosis of ACM. However, they can be useful in ACM patients with a myocarditis-like presentation (the “hot phase”) characterized by chest pains and troponin elevation, which is common in *DSP* gene mutation carriers [24,25].

CMR can be useful for the risk stratification and prediction of adverse outcomes of patients with ACM [26]. The presence of diffused LGE has been associated with an increased risk of life-threatening ventricular arrhythmias and has been incorporated into the novel ESC guidelines’ algorithm as a marker of SCD in patients with the LVEF between 35% and 50% [11].

In addition, it has been observed that patients with ALVC and those with biventricular ACM exhibited a worse prognosis compared with those with RV involvement alone [27,28]. Aquaro et al. evaluated 140 patients with a diagnosis of definite ARVC according to the 2010 task force criteria and observed that LV involvement and the LV-dominant phenotype were independent predictors of major events (i.e., the combined endpoint of SCD, appropriate ICD shock, and aborted cardiac arrest) [27].

Similarly, Wang et al. observed that in *DSP* cardiomyopathy, severe LV systolic dysfunction (an LV ejection fraction of <35%) and RV dysfunction were prognostic factors for sustained ventricular arrhythmias [29]. In addition, patients with myocardial injury (defined as chest pains, elevated troponin levels, and a normal coronary angiography) exhibited worse heart failure outcomes [29]. In contrast, the 2010 task force criteria did not identify all the affected individuals or those individuals who are more prone to experience adverse events.

Recently, the LV myocardial strain and dyssynchrony were defined as new CMR parameters using CMR-feature tracking (CMR-FT) [30]. CMR-FT shows a higher spatial resolution than echocardiographic speckle-tracking and is less operator dependent. Strain analysis by CMR-FT is useful to objectively quantify global and regional RV dysfunction and dyssynchrony in patients with AMC and adds incremental value to conventional CMR imaging [31]. In addition, the presence of significant LV dyssynchrony (defined as LV longitudinal dyssynchrony of >89.15 ms) assessed by CMR-FT was found to be an independent predictor of cardiovascular and arrhythmic events [30].

In conclusion, CMR is essential both for the diagnosis and risk stratification of patients with ACM and may be helpful in guiding management strategies.

## 4. Cardiac Nuclear Imaging

Single-photon emission computed tomography (SPECT) can allow for obtaining several imaging parameters, including LV volumes, LVEF, regional myocardial dysfunction pattern, and LV mechanical synchronization. However, its role in the diagnosis and risk stratification of patients with ALVC is still limited. Thus, it is not included in the diagnostic work-up of these patients.

Positron emission tomography (PET) has been recently introduced in the diagnostic work-up of ALVC, particularly when there is a high suspicion of an underlying specific etiology. Protonotarios et al. [32] assessed the prevalence of abnormal 18-FDG PET scans in patients with a previous diagnosis of ARVC, reporting an abnormal uptake in almost 41% of the study population. Among sixteen patients with a definite diagnosis of ACM, according to the 2010 revised task force criteria, two had pathologic evidence of cardiac sarcoidosis (CS) at the endomyocardial biopsy (EMB), and two carried a desmoplakin mutation (DSP). With the exclusion of the two patients diagnosed with CS, none of the EMBs showed signs of ongoing inflammation. However, the “patchy” nature of the inflammatory infiltrates, which are sometimes difficult to reach during a biopsy, may explain these negative findings.

Recently, the diagnostic role of 18-FDG PET in patients with arrhythmic myocarditis was evaluated [33]. Among the 75 consecutive patients evaluated, 50 (67%) showed a positive 18-FDG PET. In addition, an anteroseptal distribution pattern was found in 12/50 patients (24%), including 7/7 patients with cardiac sarcoidosis, and was associated with a greater occurrence of ventricular arrhythmias and atrioventricular blocks during the follow-up. Thus, it was suggested that, in patients with arrhythmic myocarditis, an 18-FDG PET should be useful to identify patients with worse outcomes and should raise the suspicion of cardiac sarcoidosis [33].

Furthermore, it has been postulated that the timing of an EMB could be crucial for the detection of inflammatory infiltrates, which typically may alternate the “hot phases” of active inflammation and remission [23]. Therefore, an 18-FDG PET may unveil myocardial inflammation in cases being undetected in CMR scans, particularly when the concomitant troponin elevation is identified or in the case of chest pains.

Cardiomyopathy associated with *DSP* gene variants is characterized by predominant LV involvement, recurrent episodes of myocardial injury, and a high risk of ventricular arrhythmias [34]. In a large prospective cohort study, Wang et al. [29] showed that episodes of myocardial injury were independently associated with heart failure in a multivariable analysis but not with ventricular arrhythmias.

Because both the 2010 revised task force and the Padua criteria show low sensitivity to *DSP-*related cardiomyopathy [29], an 18-FDG PET may have a role in diagnosis and risk stratification in carriers of *DSP* variants. In a recent study, patients with *DSP* cardiomyopathy had an 18-FDG PET-positive scan in 59% of cases. Of these, 70% had focal uptake and 30% diffuse uptake [35].

It has been suggested to use FDG-PET as a method to improve the assessment of patients with suspected myocarditis and for the differential diagnosis in the context of cardiomyopathies. Inflammatory heart diseases, such as myocarditis, cardiac sarcoidosis, and other immune-mediated diseases, may occasionally represent a phenocopy of ACM, resulting in misdiagnosis. Inflammation may represent the pre-phenotypic/early stage of ACM in a subgroup of patients during the “warm phase”, which is characterized by acute chest pains and/or the release of myocardial enzymes [23].

**Figure 2:**
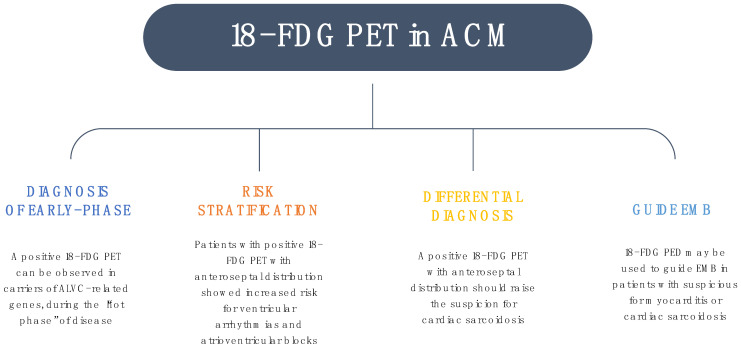
The role of 18-FDG positron emission tomography in arrhythmogenic cardiomyopathy. Abbreviations: EMB, endomyocardial biopsy.

Patients at risk have distinct clinical and genetic features, including female predominance, LV involvement, and pathogenic variants in the DSP gene. In particular, genetic testing becomes essential for the diagnosis of ACM in this cohort, suggesting the importance of genetic analysis in patients with episodes of myocarditis and at least one “red flag” [36]. Non-invasive methods, such as electrocardiography, dynamic Holter monitoring, echocardiography, and CMR, may not be sufficient to discriminate between ACM and inflammatory diseases, mimicking ACM, emphasizing the need for multimodality imaging (Figure 2).

## 5. Sudden Cardiac Death Risk Stratification

Since ALVC patients may experience life-threatening ventricular arrhythmias, it is crucial to identify high-risk patients who may benefit from an implantable cardioverter defibrillator (ICD) placement in primary prevention [11].

The new 2022 European Society of Cardiology (ESC) for the management of patients with ventricular arrhythmias and the prevention of sudden cardiac death did not provide specific recommendations for ALVC [11]. However, considering the overlapping phenotype between ALVC and HNDCM or DCM, the recommendations provided for these two conditions can also be applied to ALVC.

Multimodality imaging, coupled with clinical and electrophysiological parameters, has a key role in SCD risk stratification. As described below, several parameters evaluated through echocardiography and CMR are used to stratify ALVC patients at risk for life-threatening ventricular arrhythmias.

In particular, the LVEF guides the decision to place an ICD in primary prevention. Regardless of genetic background, ICD implantation should be considered in patients with symptomatic ALVC and an LVEF of <35% after at least 3 months of optimized medical therapy [11]. CMR represents the imaging technique of choice for SCD risk stratification in ALVC since it allows us to accurately evaluate the LVEF and to obtain information on the tissue characterization, as well as the presence and the extent of LGE, which represents a major risk factor for ventricular arrhythmias.

Moreover, risk stratification is significantly different in the presence of a specific gene mutation. Genetic testing is highly recommended in those patients who have a high probability of positive genetic testing, such as those with the DCM or HNDCM phenotype and atrioventricular conduction delay of <50 years or positive family history for cardiomyopathy or SCD [11,37,38]. In addition, in apparently sporadic ACM, a genetic analysis should also be considered [11,37].

In patients with the *LMNA* mutation, ICD implantation should be considered in patients with a 5-year estimated risk of life-threatening ventricular arrhythmias (defined as SCD- or ICD-treated or hemodynamically unstable ventricular tachycardia) ≥10% associated with the presence of non-sustained ventricular tachycardia (NSVT) or an LVEF < 50% or atrioventricular conduction anomalies [11]. The risk of life-threatening ventricular arrhythmias should be evaluated using the risk prediction model proposed by Wahbi et al., which is based on a multiparametric score including sex, non-missense *LMNA* mutations, an atrio-ventricular block, NSVT, and the LVEF calculated using echocardiography [39].

In addition, the presence of a pathogenic variant in LMNA, PLN, FLNC, and RBM20 confers additional risk for SCD. Thus, the 2020 ESC guidelines recommend an ICD implantation in patients with an LVEF between 35% and 50% and at least two risk factors, including an unexplained syncope, inducible sustained ventricular arrhythmias in programmed electrical stimulation, LGE in CMR, and pathogenic mutations in LMNA, PLN, FLNC, and RBM20 [11].

Table 2 summarizes the ACM genotype–phenotype correlations and specific recommendations for ICD implantation in the primary prevention of SCD.

Recently, a novel score (the 2019 ARVC risk score) for SCD risk stratification of patients with ACM has been proposed [40]. In particular, this score includes the following variables: sex, age, recent cardiac syncope (defined as the transient loss of consciousness and postural tone with spontaneous recovery with a likely arrhythmic origin), NSVT, the number of premature ventricular complexes on 24-h Holter monitoring, the extent of T-wave inversion on anterior and inferior leads, and the right ventricular ejection fraction [40]. In addition, the model was externally validated to confirm its reproducibility and generalizability [41]. Despite some limitations [42], the proposed model has been demonstrated to provide accurate prognostic information for patients with ARVC without a prior history of sustained ventricular arrhythmias at diagnosis. In addition, it can be useful for shared decision-making for ICD implantation.

The 2019 ARVC risk score has been shown to perform well in patients with the PKP2 pathogenic variant. In contrast, patients with the DSP pathogenic variant and in a gene-elusive cohort have demonstrated limited utility and enhancement of the need for an individualized risk approach based on the genotype [43].

## 6. Differential Diagnosis

In patients with clinical-instrumental features suggestive of ALVC, it is essential to consider, in the differential diagnosis, specific etiologies, of which diagnoses could require specific management.

### 6.1. Dilated Cardiomyopathy

DCM and ALVC share common features (i.e., LV systolic impairment and myocardial fibrosis). Thus, it is useful to underline the differences between these two conditions. DCM is characterized by relevant LV dilation and global contractility impairment, while ALVC presents commonly with normal or mildly reduced systolic function, mild or absent dilation, and regional contractile abnormalities, rare in DCM [1].

However, it is worth considering that, as recently proposed in a position paper of the ESC working group on myocardial and pericardial diseases, it has been described a disease phenotype with severe LV impairment and without LV dilation, defined as HNDCM [44]. This variant could represent the early phase of both the DCM and ALVC phenotype in the absence or in the presence of ventricular arrhythmias, respectively. These two conditions represent a continuum of a single spectrum of disease, with DCM on the one hand and ALVC on the other.

Myocardial fibrosis, evaluated as LGE in CMR, is less common in DCM, and the amount of LGE is not always associated with the severity of the LVEF and GLS impairment [45,46]. In contrast, patients with ALVC are more prone to develop myocardial fibrosis in the early phase of the disease, when the LV function is still preserved and subsequently deteriorates in the advanced stages with the progression of the scarring process [45,46]. This phenomenon reflects the different pathophysiology of the two conditions. In ALVC, the primum movens is the fibrofatty replacement, which mainly involves the subepicardial layer without compromising the subendocardial, the most responsible for LV contractility [45], while in DCM, the primary reason for LVEF depression is the myocyte contractility impairment, which induces compensatory myocardial eccentric remodeling with later developing fibrosis [47].

This different prevalence of fibrosis is responsible for potentially life-threatening ventricular arrhythmias, even in the early phases of patients with ALVC, while they generally occur in the advanced stage in those with DCM (mainly due to severe LV dysfunction), with significant implications in terms of the ICD primary prevention strategy [11].

In addition, the LGE distribution pattern is different between the two conditions. In DCM, it is common to observe a mid-wall non-ischemic LGE distribution, mainly located in the septum, whereas in ALVC, the subepicardial distribution, often located in infero and infero-lateral segments, generally occurs [47]. Furthermore, in fat-suppressed CMR sequences, a significant correspondence between the LGE distribution and intramyocardial fat was observed [47]. A specific pattern of ALVC is the subepicardial fibrosis ring, with LGE affecting the LV free wall and septum in the short axis view [14]. Finally, the identification of a disease-causing gene mutation associated with an ALVC phenotype is mandatory for the diagnosis [14].

### 6.2. Sarcoidosis

Cardiac sarcoidosis is an infiltrative cardiomyopathy characterized by the forming of non-caseating granulomas in any part of the heart, especially in the LV, resulting in contractile dysfunction, heart failure, and life-threatening arrhythmias [48]. Most patients with cardiac sarcoidosis fulfill the diagnostic criteria for ALVC, enhancing the challenge of differential diagnosis [49]. The most common CMR findings include LV or RV function impairment, LV dilatation, and typical LGE of the basal septum, rarely found in ALVC [50]. An 18-FDG PET can increase diagnostic accuracy with an uptake pattern that reflects the LGE distribution in CMR, showing active inflammation in those areas [51]. This infiltration of the basal septum may lead to atrioventricular (AV) conduction abnormalities, including AV blocks and bundle branch blocks, largely more common in cardiac sarcoidosis than in ALVC [50]. In addition, LGE distribution at the level of the RV insertion points spreading to the septum and RV (defined as “the hook sign”) represents a specific sign of cardiac sarcoidosis [50,51].

Since patients with cardiac sarcoidosis often present a systemic involvement, it is essential to search for extracardiac manifestation, such as mediastinal lymphadenopathy, which presence may help in the differential diagnosis. An endomyocardial biopsy (CMR-, PET-, or electroanatomical-mapping-guided) represents the gold standard for the diagnosis of cardiac sarcoidosis [14].

### 6.3. Myocarditis

Acute myocarditis and the LV scar caused by a previous episode of myocarditis should be considered in the differential diagnosis of patients with suspicion of ALVC.

Currently, no specific diagnostic CMR criteria exists to differentiate post-myocarditis fibrosis and ALVC, making the pedigree, clinical history, and genetic testing important tools to identify these conditions. In addition, episodes of recurrent acute myocardial injuries, with troponin elevation and acute chest pain, can be the first manifestation of ALVC (“the hot phase”), waging to misdiagnosis with acute myocarditis [52]. Thus, in patients with acute myocarditis, a comprehensive diagnostic evaluation, including family history, ECG, and CMR, is required to identify those patients with an early phase of ALVC. In particular, it has been proposed to perform genetic testing in patients with acute myocarditis and one of the following diagnostic criteria [36]:

- Family history for cardiomyopathies or SCD;
- Severe clinical presentation (e.g., severe LV systolic dysfunction or sustained ventricular tachycardia), irrespectively of age;
- Associated clinical features (echo or CMR) related to arrhythmogenic cardiomyopathy.

### 6.4. Neuromuscular Disease

Muscular dystrophies, such as Duchenne (DMD) or Becker (DMB), often has cardiac involvement with myocardial fibrosis present in 70% of patients. The assessment of LGE using CMR can be a useful tool for the early diagnosis of cardiac involvement in patients affected by muscular dystrophy [53]. The LGE pattern in these conditions is similar to that observed in ALVC, with subepicardial distribution and concomitant risk for ventricular arrhythmias [54]. Thus, family history, additional clinical features, and genetic testing are required for the diagnosis.

### 6.5. Chagas Disease

Cardiac involvement in the form of chronic myocarditis is the most important cause of mortality in patients with Chagas disease for its association with ventricular arrhythmias and SCD [55]. The pathogenesis is a diffuse inflammatory process with subsequent segmental fibrosis, more frequently localized in the posterolateral and apical walls, leading to LV systolic dilation and dysfunction [56]. In addition, the RV can be often involved.

Typical features of Chagas disease are sinus node dysfunction, atrioventricular block, and parasympathetic denervation [57], detectable by iodine-123 MBG scintigraphy, that may be associated with an increased risk of sustained ventricular tachycardia [58]. In addition, in patients with Chagas disease, it is common to observe the development of LV aneurysms, with possible thrombotic formations and its associated risk of thromboembolic stroke [56]. Besides imaging features, epidemiological factors and a serologic test are mandatory to confirm Trypanosoma Cruzi infection [55].

## 7. Conclusions

ACM describes a large spectrum of myocardial diseases with progressive fibrotic or fibrofatty replacement, responsible for the occurrence of ventricular tachyarrhythmias and ventricular dysfunction. Multimodality imaging is essential for the diagnosis of ACM and to accurately evaluate the features associated with a high risk of SCD. In addition, it plays a dominant role in the differential diagnosis of several conditions which may mimic ACM but that eventually require specific management.

## Figures and Tables

**Table 1:** 2019 International criteria for the diagnosis of arrhythmogenic left ventricular cardiomyopathy (ALVC).

Category	Diagnostic Criteria
Morpho-functional ventricular abnormalities	Minor: Global LV systolic dysfunction with or without LV dilation (assessed by echocardiography, CMR, or angiography) Minor: Regional LV hypokinesia or akinesia of the LV free wall, septum, or both
Structural myocardial abnormalities	Major: LV LGE (stria pattern) of ≥1 Bull’s Eye segment(s) (in two orthogonal views) of the free wall (subepicardial or midmyocardial), septum, or both (excluding septal junctional LGE)
Repolarization abnormalities	Minor: Inverted T waves in left precordial leads (V4–V6), in the absence of complete left bundle branch block
Depolarization abnormalities	Minor: Low QRS voltages (<0.5 mV peak to peak) in limb leads, in the absence of obesity, emphysema, or pericardial effusion
Ventricular arrhythmias	Minor: Frequent ventricular extrasystoles (>500 per 24 h), non-sustained, or sustained ventricular tachycardia with a right bundle branch block morphology (excluding the fascicular pattern)
Family history/genetics	Major: -ACM confirmed in a first-degree relative who meets diagnostic criteria -ACM confirmed at autopsy or surgery in a first-degree relative -Identification of a pathogenic or likely pathogenic ACM mutation in the patient under evaluation Minor: -History of ACM in a first-degree relative in whom it is not possible or practical to determine whether the family member meets diagnostic criteria -Premature sudden death (<35 years of age) due to suspected ACM in a first-degree relative -ACM confirmed pathologically or by diagnostic criteria in a second-degree relative

Abbreviations: ACM, arrhythmogenic cardiomyopathy; CMR, cardiac magnetic resonance; LGE, late gadolinium enhancement; LV, left ventricle.

**Table 2:** Genotype–phenotype correlations and specific recommendations for implantable-cardioverter defibrillator implantation in the primary prevention of sudden cardiac death in patients with arrhythmogenic left ventricular cardiomyopathy.

Genotype	Phenotype	Recommendations for ICD
LMNA	ALVC, DCM, muscular dystrophy, conduction delay, VT, and SCD	-SCD risk ≥ 10% and NSVT or LVEF < 50% or AV delay -LVEF < 35%
PLN	ALVC, ARVC, DCM, VT/VF	-LVEF 35–50% and ≥ 2 risk factors -LVEF < 35%
FLNC	ALVC, DCM, SCD	
RBM20	ALVC, DCM, VT	

Abbreviations: ALVC, arrhythmogenic left ventricular cardiomyopathy; ARVC, arrhythmogenic right ventricular cardiomyopathy; AV, atrioventricular; DCM, dilated cardiomyopathy; LVEF, left ventricular ejection fraction; NSVT, non-sustained ventricular tachycardia; SCD, sudden cardiac death; VF, ventricular fibrillation; VT, ventricular tachycardia.

## Data Availability

No new data were created or analyzed in the study. Data sharing is not applicable to this article.
